# Rice transcription factor OsMADS57 regulates plant height by modulating gibberellin catabolism

**DOI:** 10.1186/s12284-019-0298-6

**Published:** 2019-05-28

**Authors:** Yanli Chu, Ning Xu, Qi Wu, Bo Yu, Xingxing Li, Rongrong Chen, Junli Huang

**Affiliations:** Key Laboratory of Biorheological Science and Technology, Ministry of Education, Bioengineering College, Chongqing University, Chongqing, People’s Republic of China

**Keywords:** *OsMADS57*, Plant height, GA catabolism, Panicle exsertion, Rice

## Abstract

**Background:**

The MADS-box transcription factors mainly function in floral organ organogenesis and identity specification. Few research on their roles in vegetative growth has been reported.

**Results:**

Here we investigated the functions of *OsMADS57* in plant vegetative growth in rice (*Oryza sativa*). Knockdown of *OsMADS57* reduced the plant height, internode elongation and panicle exsertion in rice plants. Further study showed that the cell length was remarkably reduced in the uppermost internode in *OsMADS57* knockdown plants at maturity. Moreover, *OsMADS57* knockdown plants were more sensitive to gibberellic acid (GA_3_), and contained less bioactive GA_3_ than wild-type plants, which implied that *OsMADS57* is involved in gibberellin (GA) pathway. Expectedly, the transcript levels of *OsGA2ox3*, encoding GAs deactivated enzyme, were significantly enhanced in *OsMADS57* knockdown plants. The level of *EUI1* transcripts involved in GA deactivation was also increased in *OsMADS57* knockdown plants. More importantly, dual-luciferase reporter assay and electrophoretic mobility shift assay showed that OsMADS57 directly regulates the transcription of *OsGA2ox3* as well as *EUI1* through binding to the CArG-box motifs in their promoter regions. In addition, OsMADS57 also modulated the expression of multiple genes involved in GA metabolism or GA signaling pathway, indicating the key and complex regulatory role of OsMADS57 in GA pathway in rice.

**Conclusions:**

These results indicated that OsMADS57 acts as an important transcriptional regulator that regulates stem elongation and panicle exsertion in rice via GA-mediated regulatory pathway.

**Electronic supplementary material:**

The online version of this article (10.1186/s12284-019-0298-6) contains supplementary material, which is available to authorized users.

## Background

Plant height, determined by the number of elongated internodes as well as the length of internodes, is an important agronomic trait that directly affects yield potential (Yang and Hwa 2008). In “Green Revolution”, high-yielding cultivars have been created that have suitable height in rice and wheat (Khush 2001; Thomas et al. 2005). Furthermore, both the improved lodging resistance and increased total biomass result from their short stature (Sakamoto and Matsuoka 2004). Therefore, it is of great significance to clarify the signal network regulating plant height.

Gibberellin (GA) is an important phytohormone in the regulation of seed germination, flowering, leaf shape and stem elongation (Richards et al. 2001; Swain and Singh 2005). Generally, plants with a dwarf or semi-dwarf stature are deficient in GA accumulation. In the GA biosynthetic pathway, geranylgeranyl diphosphate is converted to *ent-*kaurene, which is catalyzed by *ent-*copalyl diphosphate synthase (CPS) and *ent*-kaurene synthase (KS) (Helmut Aach et al. 1997). With the catalysis of two cytochrome P450 enzymes, *ent*-kaurene oxidase (KO) and *ent*-kaurenoic acid oxidase (KAO), *ent*-kaurene is converted to GA_12_ (Helliwell et al. 2001). Then GA_12_ is converted to the bioactive GA_1_ and GA_4_ form through the 13-hydroxylation and non-13-hydroxylation pathway, respectively (Yamaguchi 2008). *CYP714B1* and *CYP714B2*, two cytochrome P450 genes, encode GA 13-oxidase that is responsible for converting GA_12_ to GA_53_, thus determining the ratio of GA_1_ and GA_4_ in rice (Magome et al. 2013). In GA homeostasis, GA intermediates can be converted into bioactive forms by GA 20-oxidase (GA20ox) and GA 3-oxidase (GA3ox), while GA 2-oxidase (GA2ox) has the opposite effect (Yamaguchi 2008; Hedden and Phillips 2000). *ELONGATED UPPERMOST INTERNODE1* (*EUI1*) encodes a putative cytochrome P450 monooxygenase that deactivates GAs through GA 16α, 17-epoxidation (Zhu et al. 2006; Luo et al. 2006; Hedden and Thomas 2012).

GA governing plant growth requires signal transduction (Xu et al. 2014). *GIBBERELLIN INSENSITIVE DWARF1* (*GID1*) encodes a soluble receptor for perceiving GA (Ueguchi-Tanaka et al. 2005). Binding of GA to GID1 causes GID1 conformation change, stimulating the interaction of GID1 and SLENDER1 (SLR1), a DELLA family protein acting as the GA signaling repressor (Tong et al. 2014; Hedden and Sponsel 2015). The GA-GID1-DELLA complex is recognized and polyubiquitylated by E3 ubiquitin ligase complex, and subsequently degraded through the 26S proteasome pathway, thereby stimulating GA response (Daviere and Achard 2016; Feng et al. 2008; Fleet and Sun 2005). Mutations in the components involved in GA metabolism as well as GA signaling pathway significantly affect plant growth (Thomas et al. 2005). *d18* and *sd1* are loss-of-function mutants that result from mutations in *OsGA3ox2* and *OsGA20ox2*, respectively, and exhibit remarkable dwarf or semi-dwarf phenotype (Sakamoto et al. 2004). In addition, overexpression of the *EUI1* greatly reduces plant height (Zhang et al. 2008). Accordingly, rice *slender*-type mutant (*slr1*) contains a loss-of-function mutation in the *SLR1* gene, and thereby shows a significant increase in plant height (Ikeda et al. 2001).

A number of transcription factors are involved in the development of plant height through regulating GA metabolism. In rice, OsNAC2 negatively regulates plant height by suppressing the expression of *KO2* that is involved in GA biosynthesis (Chen et al. 2015). The YABBY family members such as OsYABBY1 and OsYABBY4 can reduce bioactive GA levels by repressing the expression of *OsGA3ox2* and *OsGA20ox2*, respectively, thus reducing plant height. OsWOX3A directly represses the transcription of *KAO* that encodes GA biosynthetic enzyme. Thus overexpression of *OsWOX3A* leads to severe dwarfism in rice plants (Cho et al. 2016). Homeodomain-leucine zipper (HD-ZIP) transcription factor HOX12 activates the expression of *EUI1* by binding to its promoter sequence, thus reducing plant height and panicle exsertion (Gao et al. 2016); overexpression of rice *HOMEOBOX4 (Oshox4)* also leads to a semi-dwarf phenotype by inhibiting cell elongation in stems (Dai et al. 2008; Zhou et al. 2014). Additionally, OsEATB, an APETALA2 (AP2)/Ethylene-Responsive Element Binding Factor (ERF), can reduce rice plant height by down-regulating the transcription of *OsCPS2* that encodes GA biosynthetic enzyme (Qi et al. 2011). These transcription factors form a regulatory system to participate in the development of plant height.

MADS-box proteins are an important class of transcription factors that can bind to CArG motifs in the promoter regions of the target genes (Tang and Perry 2003; Huang et al. 1996), which has a vital significance in plant growth and development. In *Arabidopsis thaliana*, AGL15 (AGAMOUS-Like 15) increases production of somatic embryos via activating the expression of *AtGA2ox6* (Wang et al. 2004). In rice, suppression of short vegetative phase (SVP)-group MADS-box genes such as *OsMADS22*, *OsMADS47* and *OsMADS55* that play the negative regulatory role in BR signaling, results in increased lamina joint inclination (Lee et al. 2008; Duan et al. 2006). OsMADS3 is involved in regulating flower meristem determinacy and pistil development (Yasui et al. 2017). In addition, OsMADS6 regulates flower morphogenesis by activating the expression of *FACTOR OF DNA METHYLATION LIKE 1* (*OsFDML1*) that is involved in maintaining normal floral organ identity (Tao et al. 2018). *OsMADS57* mainly expresses in leaves (Puig et al. 2013), and the physical interaction between OsTB1 and OsMADS57 can alleviate the transcriptional inhibition of OsMADS57 on *D14*, thereby inhibiting the production of axillary buds and reducing the number of tillers (Yao et al. 2016; Arite et al. 2009; Guo et al. 2013a). In addition, only under the cold stress, OsMADS57 can activate the expression of *OsWRKY94* by directly binding to the promoter region of *OsWRKY94* to enhance the chilling tolerance of rice plants (Chen et al. 2018). Although MADS-box proteins play a variety of functions in plant growth and development, little is known about the function of OsMADS57 regulating plant height in rice. Here, we found *osmads57* mutants show a semi-dwarf phenotype that can be rescued by application of GA_3_. Further research demonstrated that OsMADS57 binds to the promoter regions of *OsGA2ox3* as well as *EUI1*, respectively, thus inhibiting their expression. These results show that *OsMADS57* affects plant growth by regulating gene expression of GA metabolism, which reveals a molecular mechanism that OsMADS57 controls plant height in rice.

## Results

### *osmads57* mutants display a semi-dwarf phenotype

**Fig. 1:**
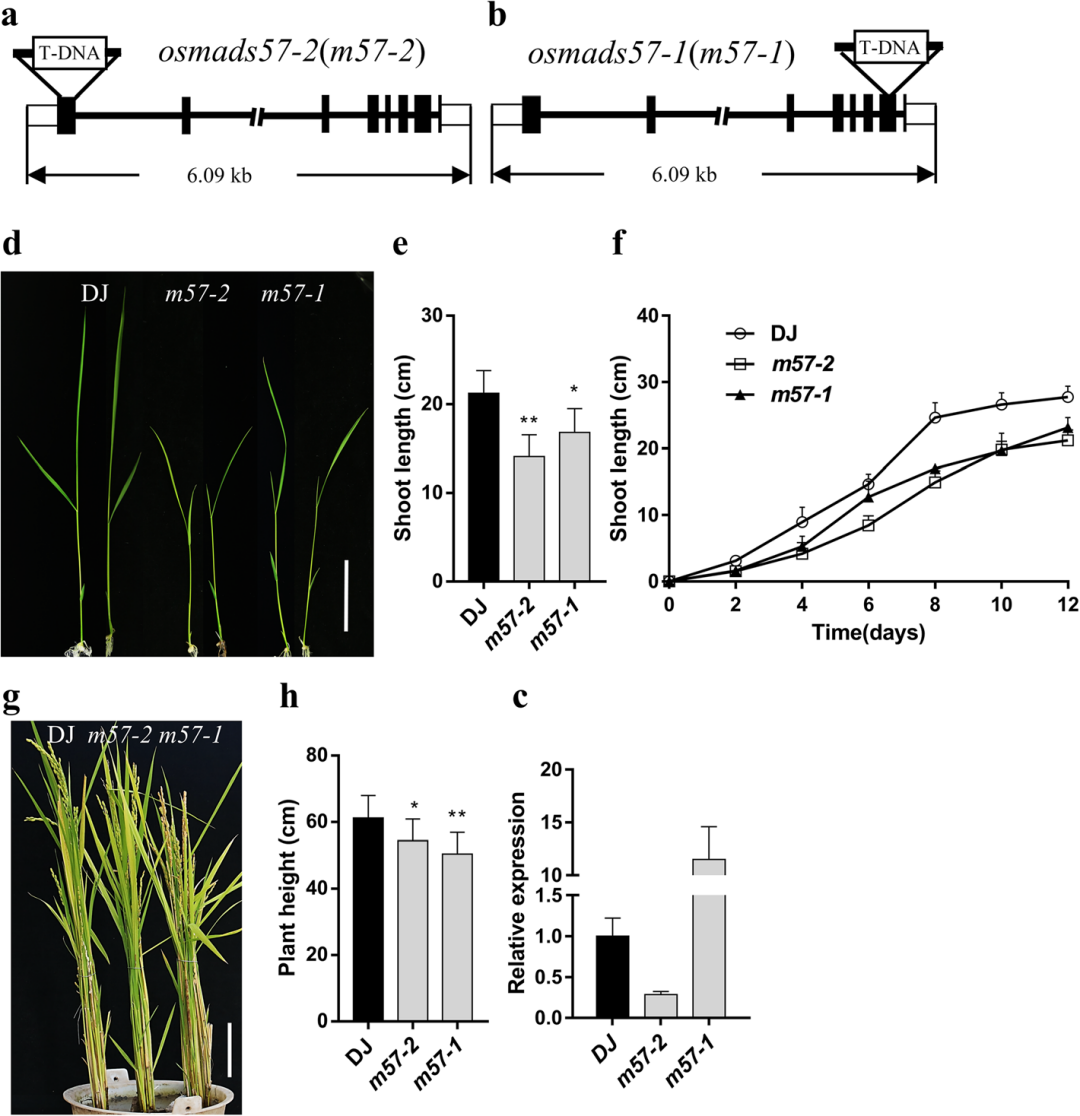
Phenotypes of the *m57–2* and *m57–1* mutants. **a** and **b** Schematic diagram indicating the T–DNA insertion site in genomic region in *m57–2* (**a**) and *m57–1* (**b**). **c** Transcript levels of *OsMADS57* in mutants and wild type lines by Q–PCR analysis. **d** The 10-day-old wild type and *osmads57* plants grown in standard 1/2 MS medium. Bars = 5 cm. **e** Shoot length of 10-day-old wild type and *osmads57* seedlings. **f** Shoot length of wild type and *osmads57* plants grown in standard 1/2 MS medium for different days. **g** Morphology of wild type and *osmads57* plants at maturity. Bars = 10 cm. **h** Plant height of wild type and *osmads57* plants at maturity. DJ, wild type; *m57–2*, *osmads57–2*; *m57–1*, *osmads57–1*. Three independent experiments were conducted with similar results. The data are means± SD (*n* = 30). Error bars indicate SD. The statistical significance of the measurements was determined by Student’s *t*-test. Asterisks indicate the significant difference between *osmads57* and wild type (*t*–test, * *P* < 0.05, ** *P* < 0.01 or *** *P* < 0.001)

The rice gain-of-function mutant *osmads57–1* (*m57–1*) shows increased tiller numbers and improved chilling tolerance (Guo et al. 2013a; Chen et al. 2018), whereas the loss-of-function mutant *osmads57–2* (*m57–2*) exhibits weakened chilling tolerance (Chen et al. 2018). The *m57–2* line is a knockdown mutant in which the T-DNA was inserted in the 5′ terminus of *OsMADS57*, + 61 from ATG (Fig. 1a, Additional file 1: Figure S1a-c). In *m57–1* line, the T-DNA was inserted in 3′ terminus of *OsMADS57* (Fig. 1b), forming truncated protein (Guo et al. 2013a). Further, quantitative PCR (Q-PCR) analysis exhibited the transcript levels of *OsMADS57* were significantly reduced in *m57–2*, whereas remarkably increased in *m57–1* (Fig. 1c). Intriguingly, both *m57–2* and *m57–1* showed a semi-dwarf phenotype in rice plants at seedling stage as well as at maturity (Fig. 1d and g). Compared to that in wild type, the shoot length was significantly reduced in the mutant seedlings, with 34% less in *m57–2* and 21% less in *m57–1* than wild type, respectively (Fig. 1d-f). Expectedly, the plant height of *m57–2* and *m57–1* was also significantly reduced compared to that of wild type at maturity, with 12% less in *m57–2* and 18% less in *m57–1* than wild type, respectively (Fig. 1g and h). At maturity, *m57–1* plants exhibited more tillers (Fig. 1g), which was consistent with the previous report (Guo et al. 2013a). GA is also involved in flowering and seed germination (Guo et al. 2013b; Olszewski et al. 2002). We found that *m57–2* plants showed the phenotype of late flowering (Additional file 1: Figure S1d and e). In addition, *m57–2* also exhibited delayed seed germination compared to that of wild type, although almost all seeds germinated on day 5 (Additional file 1: Figure S1f). These investigations indicate that abnormal expression of *OsMADS57* affects plant height, which might be caused by GA deficiency and/or defect in GA response.

### Abnormal expression of *OsMADS57* affects internode cell length

**Fig. 2:**
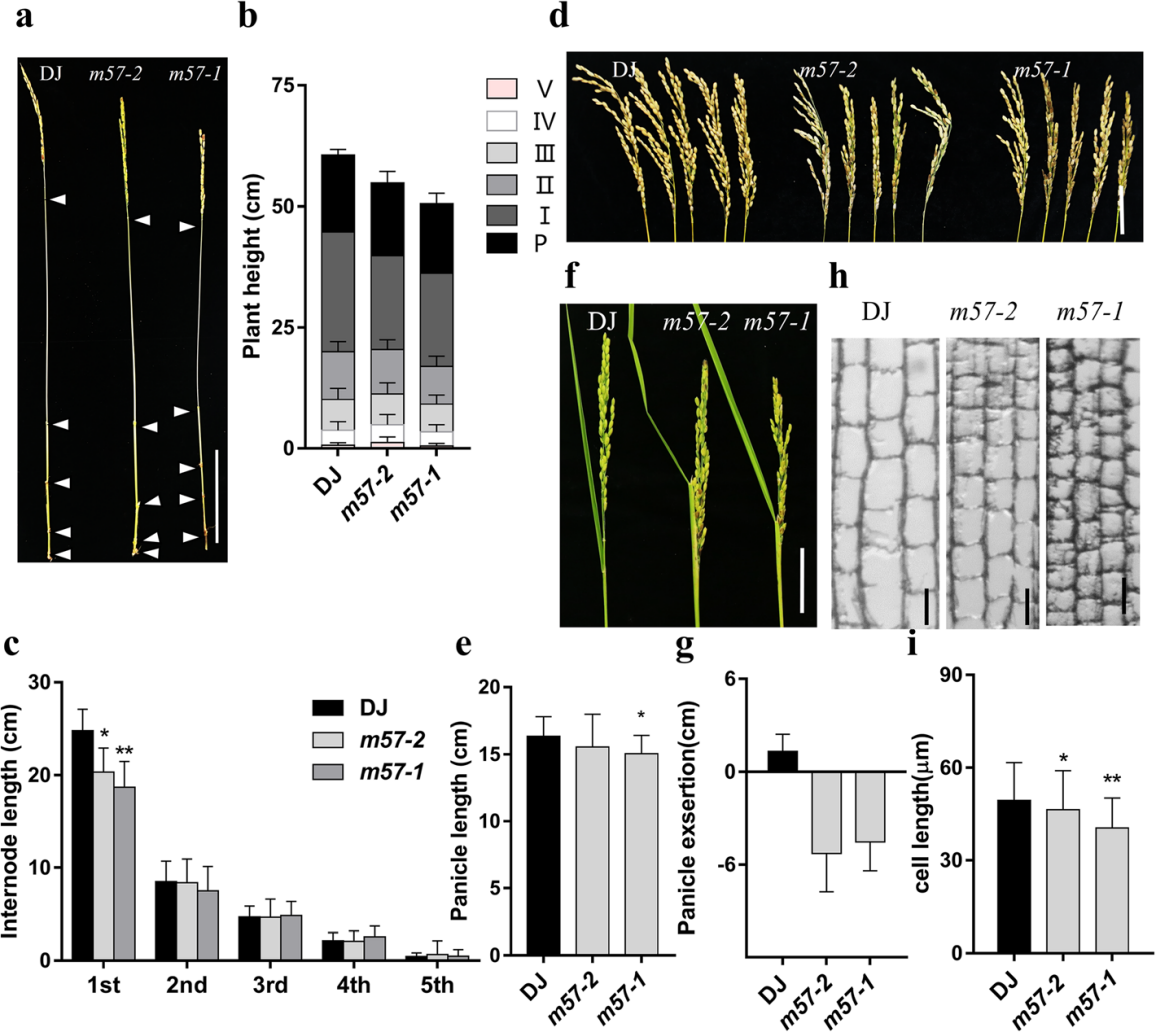
Characterization of *osmads57* plants. **a** Plant height of wild type and mutants*.* Bars = 10 cm. **b** Plant height and the length of its components of wild type and mutants. P, panicle; I, the uppermost internode; II, III, IV, the second, third, and fourth internodes counted from the uppermost internode, respectively. **c** Individual internode lengths of wild type and mutants. **d** The phenotype of panicles. Bars = 5 cm. **e** Panicle length of wild type and mutants at maturity. **f** Panicle exsertion of wild type and mutants. Bars = 2 cm. **g** Measurement of panicle exsertion of wild type and mutants. **h** Longitudinal sections of the uppermost internodes of wild type and mutants. Bars = 50 μm. **i** Quantitation of cell length of the uppermost internode of wild type and mutants. DJ, wild type; *m57–2*, *osmads57–2*; *m57–1*, *osmads57–1*. Three independent experiments were conducted with similar results. The data are means± SD (*n* = 15). Error bars indicate SD. The statistical significance of the measurements was determined by Student’s *t*-test. Asterisks indicate the significant difference between *osmads57* and wild type (*t*–test, * *P* < 0.05, ** *P* < 0.01 or *** *P* < 0.001)

The plant height is determined by the length of each internode and the total number of internodes (Wang and Li 2005; Yang and Hwa 2008). Then we compared the internode length of mutants with that of wild-type plants. Statistical analysis showed that the uppermost internode length of *m57–2* and *m57–1* was drastically reduced compared to that of the wild type (Fig. 2a-c). We also found that the panicle length of *m57–1* was greatly reduced than that in wild type, but the panicle length of *m57–2* was not significantly altered (Fig. 2d and e). Thus, our results indicate that the semi-dwarf phenotype of two mutants is caused by the reduced internode length. In addition, there was no great significance in the flag leaf length between mutants and wild type (Additional file 2: Figure S2a and b). It is notable that, different from panicles completely out of the leaf sheath in wild type, panicles of mutants were only partly out of the leaf sheath and even *m57–2* plants displayed severer panicle enclosure phenotype than *m57–1* plants at heading stage (Fig. 2f and g). In addition, we found the seed setting rate in *m57–2* and *m57–1* was also reduced (Additional file 2: Figure S2c), with many shrunken grains (Additional file 2: Figure S2d). Nevertheless, the grain size between mutants and wild type was not significantly different (Additional file 2: Figure S2d). Together, our data show that the mutants are defective in the elongation of the uppermost internode. Internode length is mostly influenced by the cell length and cell number (Dai et al. 2007; Gao et al. 2016). To explore whether the reduction of internode length in mutant lines is caused by abnormal cell elongation or/and cell proliferation, we compared the longitudinal sections of the elongation zone in the uppermost internode of wild type, *m57–2* and *m57–1* plants. Microscopic observation revealed that the cell length in the uppermost internode of *m57–2* and *m57–1* significantly reduced compared to that of the wild type (Fig. 2h and i), respectively. These data indicate that the abnormal expression of *OsMADS57* impairs the cell elongation of the uppermost internode.

### Exogenous GA eliminates the semi-dwarf stature of *osmads57* mutants

**Fig. 3:**
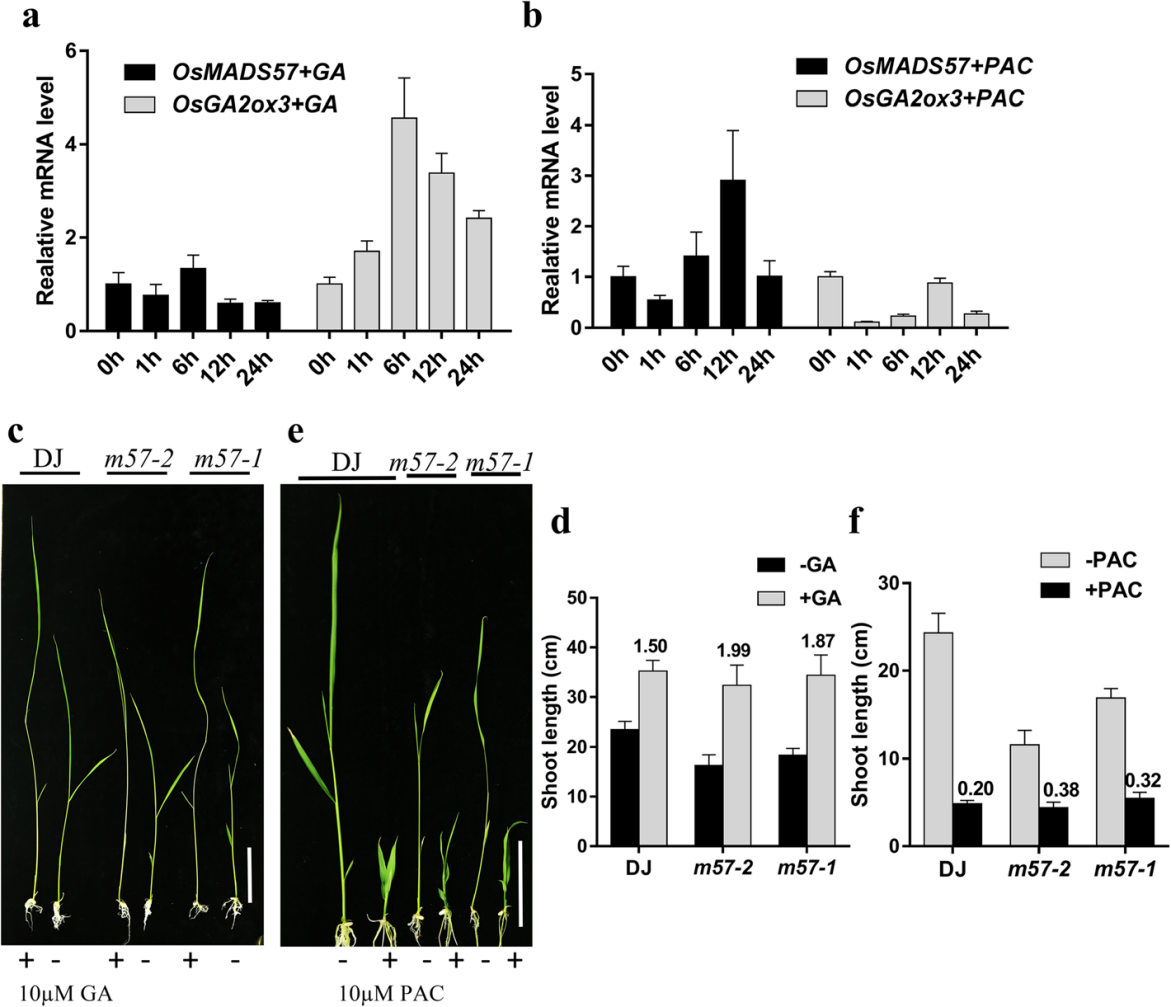
Response of *m57–2* and *m57–1* mutants to GA and PAC. **a** and **b** 50 μМ GA_3_ (**a**) or 10 μМ paclobutrazol (**b**) treatment suppressed and enhanced the expression of *OsMADS57*, respectively. *OsGA2ox3* was used as a positive control. **c** Semi-dwarf phenotype of *m57–2* and *m57–1* mutants can be rescued by GA_3_. The mutants were treated with exogenous 10 μМ GA_3_ for 10 d. Bars = 5 cm. **d** Shoot length of seedlings before and after 10 μМ GA_3_ treatment for 10 d. **e** Seedling morphology of mutants and wild-type plants grown with or without 10 μМ PAC for 10 d. Bars = 5 cm. **f** Shoot length of seedlings before and after 10 μМ PAC treatment for 10 d. Numbers above the bars indicate the elongation ratio of seedlings after treatment (the shoot length after treatment divided by the shoot length before treatment). DJ, wild type; *m57–2*, *osmads57–2*; *m57–1*, *osmads57–1*. Three independent experiments were conducted with similar results. The data are means± SD (*n* = 15). Error bars indicate SD

Phytohormones play an important role in regulating the growth and development of plants. GA, one of the important plant hormones, generally regulates cell elongation and determines plant height (Thomas et al. 2005). To explore whether GA modulates the expression of *OsMADS57*, the wild-type plants were treated with GA_3_ and paclobutrazol (PAC), a GA biosynthesis inhibitor, for transcriptional analysis. *OsGA2ox3*, up-regulated by GA_3_ and down-regulated by PAC (Boden et al. 2014; Sakai et al. 2003; Puig et al. 2013; Magome et al. 2013), was used as a positive control for the experiment. Q-PCR showed that the expression of *OsMADS57* was suppressed by GA_3_, whereas up-regulated by PAC (Fig. 3a and b). GA, acting as a signal molecule, has a major function in signal transduction, and the malfunction in biosynthesis or signaling pathway of GA can result in typical dwarf or semi-dwarf phenotype (Thomas et al. 2005). To investigate whether the semi-dwarf phenotype of mutants is caused by GA deficiency or defects in GA signaling, we investigated the response of *m57–2* and *m57–1* to exogenous GA_3_. The application of exogenous GA greatly promoted the growth of two mutant seedlings, which exhibited a slender phenotype, and the shoot length of *m57–2* and *m57–1* was fully rescued compared to that of wild type without GA application (Fig. 3c), although the shoot length of two mutant plants was slightly shorter than that of wild-type plants under GA treatment (Additional file 3: Figure S3a). For comparison, we calculated the elongation ratio of seedlings under 10 μМ GA_3_ treatment compared with the untreated plants. Both *m57–2* and *m57–1* had the elongation ratio of 1.99 and 1.87, respectively. However, the ratio for the wild type was 1.50, which was lower than that of the two mutants (Fig. 3d). Thus *m57–2* and *m57–1* mutants were more sensitive to GA_3_ than wild type. Furthermore, we also treated seedlings with PAC. We found that the growth of mutants and wild type was all inhibited, with thick root and wide leaf phenotypes (Fig. 3e, Additional file 3: Figure S3b). The elongation ratio of seedlings under 10 μМ PAC treatment compared with the untreated plants was calculated. We found *m57–2* and *m57–1* had the higher elongation ratio compared with wild type, with the ratio of 0.38:0.20 and 0.32:0.20 (Fig. 3f), respectively. Together, these data indicate that both *m57–2* and *m57–1* have enhanced GA_3_ sensitivity and reduced PAC sensitivity, which implies that GA signaling pathway in *osmads57* mutant plants is not impaired.

### *OsMADS57* alters the transcription of genes involved in GA metabolism

**Fig. 4:**
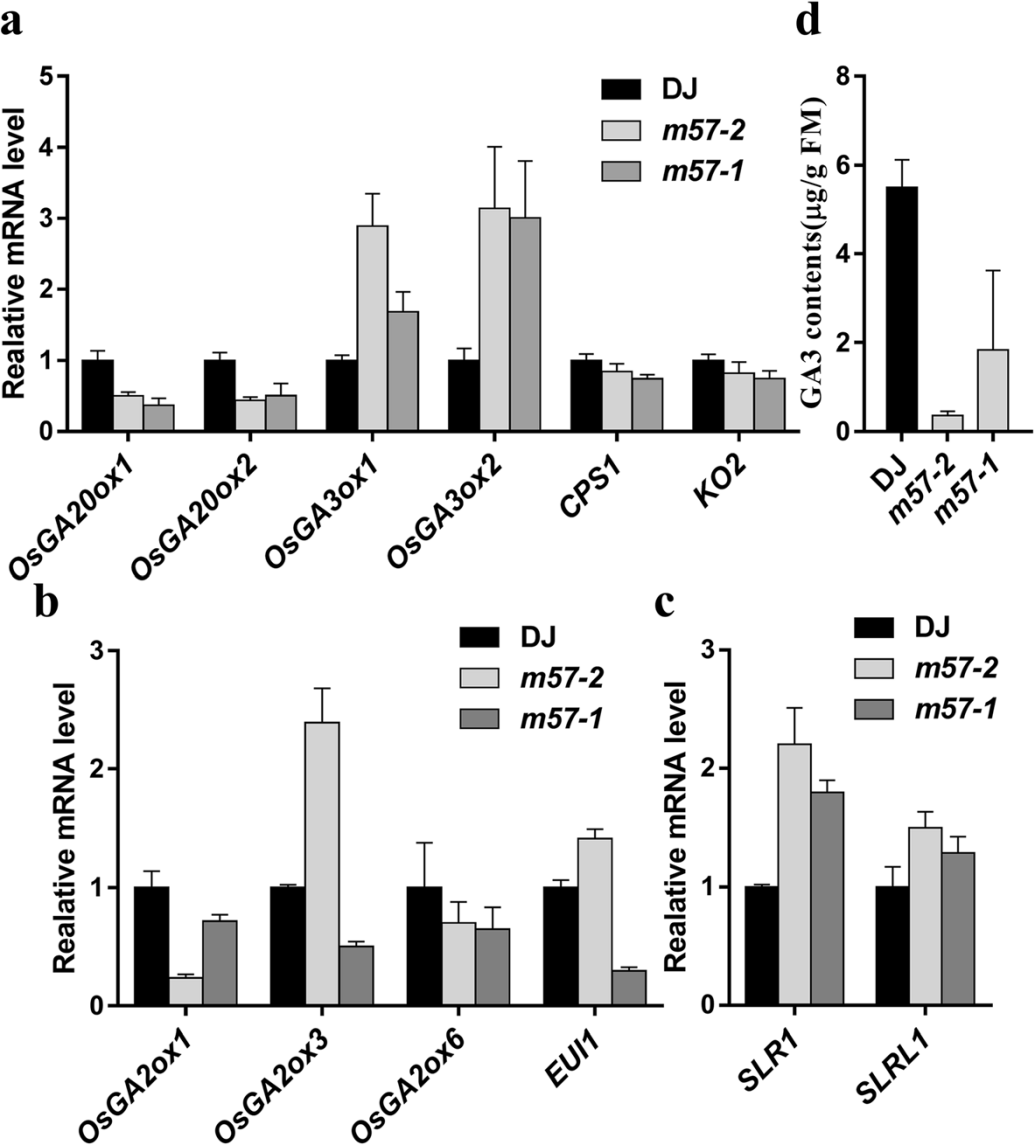
OsMADS57 regulate the expression of GA metabolic genes. **a**-**c** Altered expression of genes involved in GA metabolism (**a** and **b**) and signaling (**c**). Expression of genes involved in GA metabolism and signaling was analysed by Q-PCR in wild type and mutants lines. The PCR signals were normalized with that from *actin1* transcripts. Transcript levels from wild type were set at 1. **d** Quantification of GA_3_ in four-leaf stage seedlings. DJ, wild type; *m57–2*, *osmads57–2*; *m57–1*, *osmads57–1*. Three independent experiments were conducted with similar results. Data are means ± SE (*n* = 10). Error bars indicate SE

To determine whether the inhibition of internode elongation in *m57–2* and *m57–1* plants is caused by reduced contents of bioactive GAs, we firstly investigated the expression of genes encoding GA metabolic enzymes by Q-PCR analysis. We found that the transcript levels of genes involved in GA biosynthesis such as *OsGA20ox1, OsGA20ox2, CPS1* and *KO2* were down-regulated in *m57–2* and *m57–1* plants compared with that in wild type (Fig. 4a). Next we compared the expression of genes encoding GA catabolic enzymes in mutants and wild type (Fig. 4b). We found that both *m57–2* and *m57–1* exhibited reduced transcription levels of *OsGA2ox1* and *OsGA2ox6*. In contrast, the mRNA levels of *OsGA2ox3* as well as *EUI1* were increased in *m57–2* plants but reduced in *m57–1* plants.

The response of plants to GA is not only determined by GA accumulation, but by GA signaling (Ueguchi-Tanaka et al. 2005; Sakamoto et al. 2004). Altered the expression of genes involved in GA signaling pathway also affects the GA response (Ikeda et al. 2001). SLR1 and SLRL1, as the central suppressors of GA signaling pathway, play an important role in GA response (Fukao and Bailey-Serres 2008). Overexpression of *SLRL1*, homologous to *SLR1*, induces a dwarf phenotype in rice (Itoh et al. 2005). Thus, we examined the transcript levels of *SLR1* and *SLRL1* in wild type, *m57–2* and *m57–1*. Intriguingly, the results showed that the transcription of *SLR1* and *SLRL1* was up-regulated in two mutant plants (Fig. 4c). We speculated that the down-regulated expression of genes involved in GA biosynthesis led to reduced accumulation in GA levels in *m57–2* and *m57–1*. To confirm this hypothesis, endogenous levels of bioactive GA were measured. We found that the GA_3_ content in *m57–1* plants decreased by more than half compared with that of the wild type, while the GA_3_ content in *m57–2* plants decreased more severely (Fig. 4d). Taken together, the above data show that defect in the stem elongation in *m57–2* and *m57–1* is caused by the reduced GA levels.

### OsMADS57 regulates the transcription of *OsGA2ox3* and *EUI1* by interacting with their promoters

DELLA protein is the repressor of GA signaling, and inhibition of the function of DELLA protein triggers GA responses (Itoh 2002; Hirano et al. 2012; Ikeda et al. 2001). DELLA, lacking a DNA-binding domain, regulates downstream genes via interacting with other proteins (Yoshida et al. 2014; Fukazawa et al. 2014; Chen et al. 2017). To investigate whether OsMADS57 interacts with SLR1, we conducted yeast two-hybrid assay. We found that there was no direct physical interaction between OsMADS57 and SLR1 (Additional file 4: Figure S4).

**Fig. 5:**
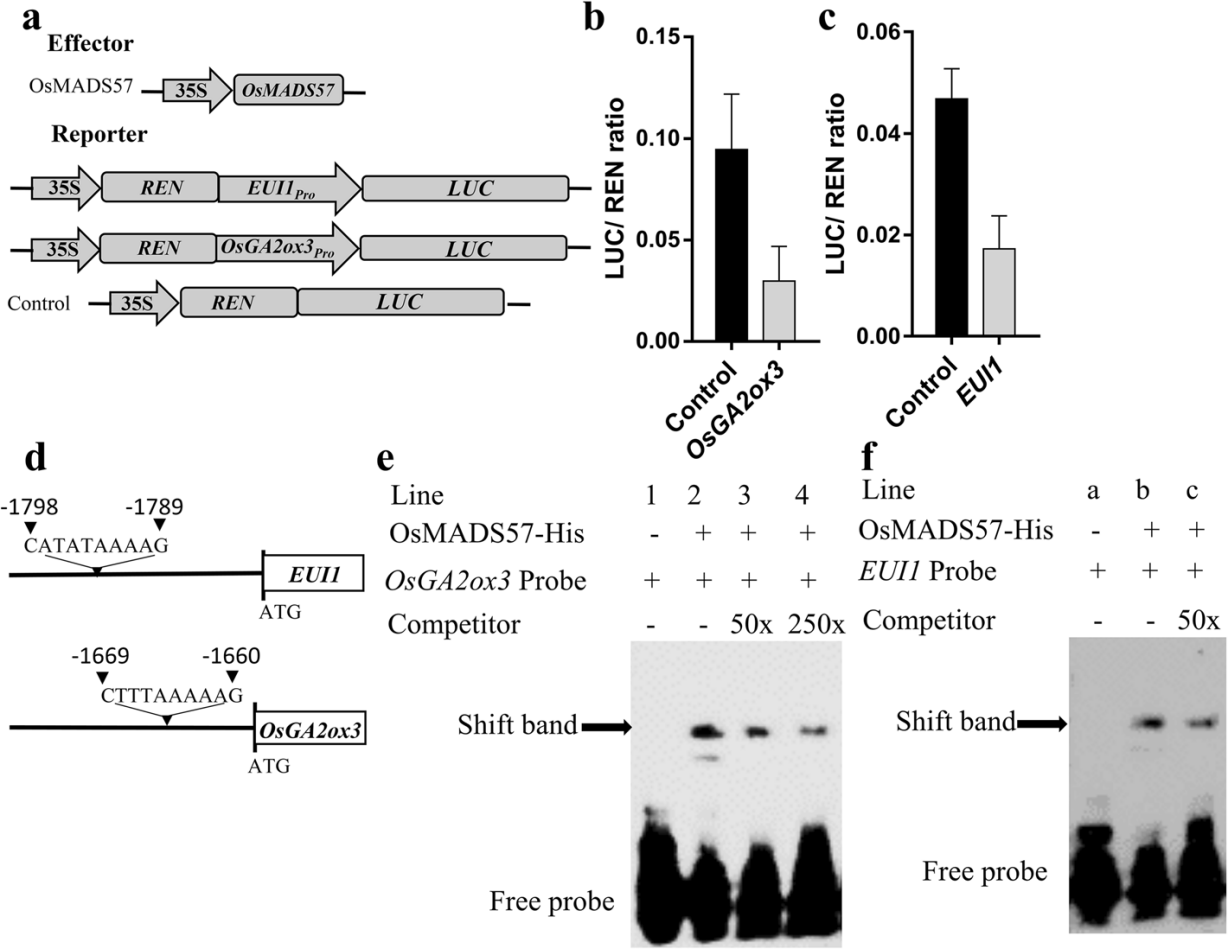
OsMADS57 directly regulates the expression of *OsGA2ox3* and *EUI1* by binding to the promoter regions of *OsGA2ox3* and *EUI1*, respectively. **a** Schematic diagrams of the effector and reporter constructs used in the dual-luciferase reporter assays. **b** and **c** The transient transactivation assay in rice protoplasts. **d** Schematic diagram of *OsGA2ox3* and *EUI1* promoter regions showing the CArG-box. **e** and **f** EMSA showed that OsMADS57 bound to CArG-box in the promoter regions of *OsGA2ox3* (**e**) and *EUI1* (**f**). Three independent experiments were conducted with similar results. Data are means± SD (*n* = 9). Error bars indicate SD

It was shown that the *eui1* mutant shows the elongated uppermost internodes and elevated levels of bioactive GA compared with wild type (Luo et al. 2006; Zhang et al. 2008; Zhu et al. 2006); while overexpression of *EUI1* caused extreme dwarfism and reduced the content of bioactive GA (Hedden and Thomas 2012). Q-PCR analysis showed that the transcript levels of *OsGA2ox3* as well as *EUI1* were increased in *m57–2* plants but reduced in *m57–1* plants (Fig. 4b). As a transcription factor, OsMADS57 binds to CArG-box motifs in the promoter regions to regulate the transcription of target genes (Chen et al. 2018; Wang et al. 2004). Thus, to investigate whether OsMADS57 regulates the transcription of *OsGA2ox3* and *EUI1*, we performed a dual-luciferase reporter assay. The promoter sequence containing the CArG motif was constructed into the reporter vector, which contains *35S*_*Pro*_*:REN* as an internal control, and the firefly luciferase reporter driven by the *OsGA2ox3* or *EUI1* promoter. *OsMADS57* was expressed in the effector vector (Fig. 5a). The reporter and effector were co-transformed into rice protoplasts. After transiently expressed in rice protoplasts, the LUC and REN activities were then measured and the LUC activity was normalized to REN activity. We found that co-expression of *35S:OsMADS57* and *OsGA2ox3*_*Pro*_*:LUC* significantly reduced the LUC:REN ratio, and the LUC activity was 32% of that in the control (Fig. 5b). As to *EUI1*, the LUC activity was also repressed by co-transformation of *35S:OsMADS57* and *EUI1*_*Pro*_*:LUC*, and the LUC activity was 37% of that in the control (Fig. 5c). LUC activity analysis showed that OsMADS57 inhibited the expression of *OsGA2ox3* and *EUI1*, indicating that OsMADS57 is a negative regulator of *OsGA2ox3* and *EUI1.* To further investigate whether OsMADS57 can bind to DNA motifs in the promoter region of *EUI1* or *OsGA2ox3*, electrophoretic mobility shift assays (EMSAs) were used with His-OsMADS57 protein. We identified CArG motifs by surveying the promoter regions of *EUI1* and *OsGA2ox3.* As shown in Fig. 5d, the potential OsMADS57-binding CArG-box sites, CATATAAAAG at − 1789 to − 1798 bp from ATG position of *EUI1* and CTTTAAAAAG at − 1660 to − 1669 bp from ATG position of *OsGA2ox3*, were found*.* As shown in Fig. 5e, shifted band was observed when probes containing CTTTAAAAAG in the *OsGA2ox3* promoter region were incubated with OsMADS57 protein (lane 2). By contrast, no shift band was detected when the sample contains the probe alone (lane 1). The binding of OsMADS57 protein to the labeled probe was competed by unlabeled probe (lane 3; 4). As we expected, shifted band was also observed when probes containing CATATAAAAG in the *EUI1* promoter region were incubated with OsMADS57 protein (lane b) (Fig. 5f). On the contrary, no shift band was detected when the sample only contains the probe (lane a). As expected, the binding band can be competed by unlabeled probe (lane c). Thus, our results indicate that *OsMADS57* is an upstream transcriptional regulator of *OsGA2ox3* as well as *EUI1*.

### mRNA expression analysis of mutant plants

**Fig. 6:**
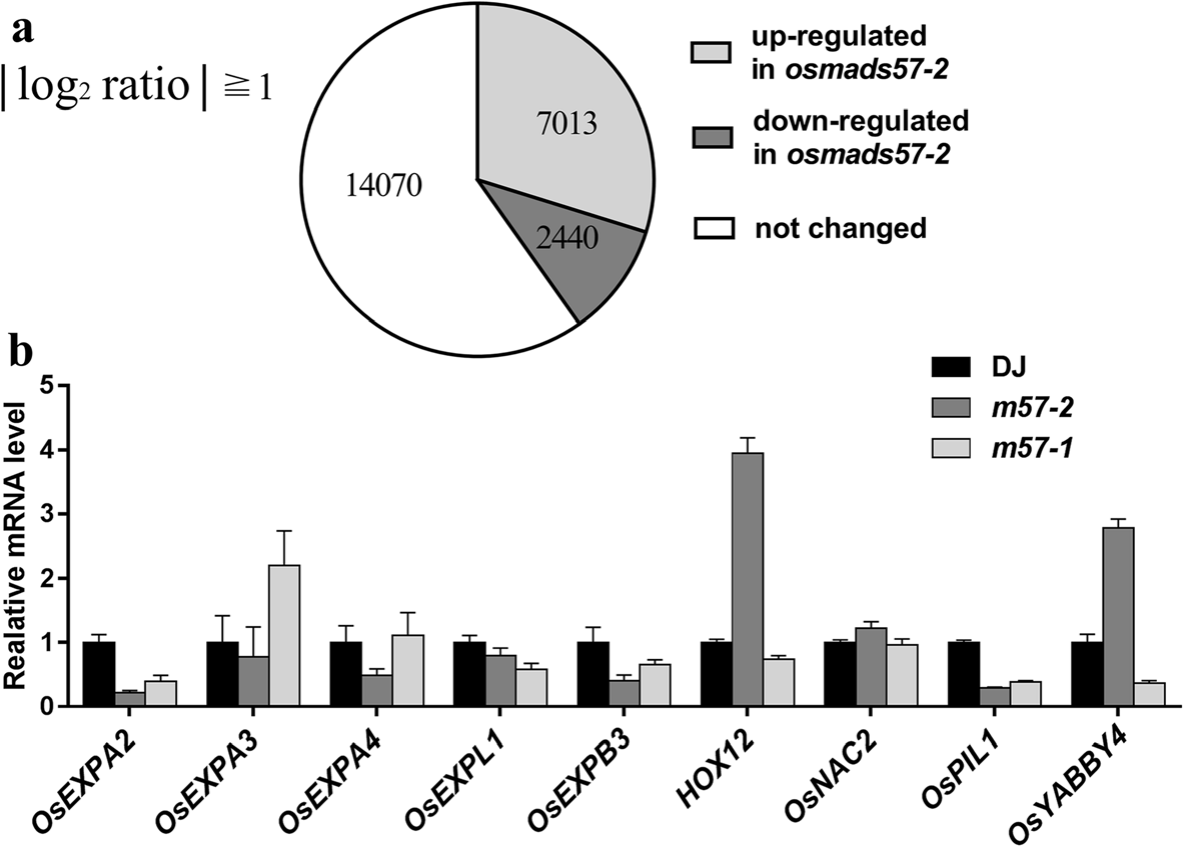
Expression analysis of genes involved in GA metabolism and cell wall loosening. **a** Expression changes of genes in *m57–2* seedlings from the RNA-seq analysis. **b** Expression of genes involved in GA metabolism and cell wall loosening in mutants and wild-type lines was analysed by Q-PCR. DJ, wild type; *m57–2*, *osmads57–2*; *m57–1*, *osmads57–1*. Three independent experiments were conducted with similar results. Data are means± SE (*n* = 12). Error bars indicate SE

Based on the semi-dwarf phenotype of mutants, we conducted RNA sequencing (RNA-seq) using shoots of *m57–2* and wild type to analyze the genes involved in plant growth and development. The transcriptome analysis showed that 7013 genes were up-regulated and 2440 genes were down-regulated in *m57–2* compared with that in wild type (Fig. 6a, Additional file 6: Data Set S1). Among the changed genes, we found that the expression of genes involved in GA metabolism and cell wall loosening had changed greatly (Table 1). The expression of *HOX12* was significantly induced in *m57–2* plants. Accordingly, reducing the expression of *HOX12* promotes panicle exsertion in rice plants (Gao et al. 2016). The transcript levels of *OsYABBY4* in *m57–2* plants were 6.40-fold greater than that in wild-type plants. It was shown that overexpression of *OsYABBY4* reduces the plant height through repressing the expression of *OsGA20ox2* (Yang et al. 2016). In addition, cell wall loosening-related genes (*EXPs*), which influence plant height through wall extension (Shcherban et al. 1995), were also altered greatly. The promoter region of *OsEXPA4* that is involved in expansin synthesis contains GA-responsive elements (GARE), and *OsEXPA4* can be induced by GA (Lee et al. 2001). However, the expression of *OsEXPA4* was found to be 2.02-fold less in *m57–2* plants than that in wild-type plants. To further verify the gene expression was changed, we performed Q-PCR analysis. The results showed that expansin genes were down-regulated in mutant plants (Fig. 6b). Increased transcription levels of *HOX12* and *OsYABBY4* were detected in *m57–2* plants, which was consistent with the results of RNA-seq (Table 1). Our results suggest that *OsMADS57* affects plant height by regulating the expression of genes involved in GA metabolism and extension of the plant cell wall.

**Table 1:** Expression of genes involved in GA metabolism and cell wall loosening in *osmads57–2* plants

gene	Change(Log2 ratio)	Annotation
Os04g0228400	−4.75	OsEXPA1
Os01g0823100	−2.06	OsEXPA2
Os05g0477600	−2.02	OsEXPA4
Os06g0725300	−1.69	OsEXPL4
Os10g0555900	−1.94	OsEXPB3
Os04g0552200	−3.00	OsEXPB5
Os04g0598300	2.58	APO2
Os04g0610400	2.16	OsAP2–39
Os03g0782500	−3.20	OsPIL1
Os03g0198600	7.45	HOX12
Os02g0643200	6.40	OsYABBY4
Os04g0460600	2.16	OsNAC2
Os06g0275000	−2.88	OsHd1

Changes in gene expression based on the RNA-seq data

## Discussion

### OsMADS57 controls plant height via GA homeostasis

Rice plant height, an important agronomic trait, largely affects crop yields (Yang and Hwa 2008). Many transcription factors are involved in regulating plant height (Chen et al. 2015; Todaka et al. 2012; Qi et al. 2011). Increased expression of *SHORT* and *SOLID CULM*, the rice homologue of *Arabidopsis LFY*, drastically reduces the plant height (Wang et al. 2017). In this work, we demonstrated that the MADS-box transcription factor OsMADS57 directly suppressed the expression of *OsGA2ox3* and *EUI1*, resulting in reduced levels of bioactive GA and plant height in *m57–2*. The expression of *OsMADS57* was induced by PAC but repressed by GA_3_ (Fig. 3a and b), which was consistent with previous research (Puig et al. 2013). Both the loss-of-function mutant *m57–2* and gain-of-function mutant *m57–1* displayed semi-dwarf phenotype in seedlings, and the semi-dwarf phenotype can be rescued by applying GA_3_ (Figs. 1 and 3). These results indicate that *OsMADS57* is associated with GA-mediated regulatory pathway. Endogenous GA levels are modulated by the expression of genes involved in GA biosynthesis and deactivation, and also fine-tuned by feedback control of GA metabolism (Yamaguchi 2008; Hedden and Thomas 2012; Fukazawa et al. 2017; Boden et al. 2014; Thomas et al. 2005; Fukazawa et al. 2011). The transcript levels of GA biosynthesis genes were reduced in *m57–2* and *m57–1* plants, resulting in less bioactive GA accumulation than wild type (Fig. 4a and d). Interestingly, the transcription of *OsGA3ox1* and *OsGA3ox2* was up-regulated in both mutant plants, which may account for a feedback mechanism due to the reduced levels of bioactive GA in both mutants. Thus, it was likely that two mutants showed reduced transcription of *OsGA2ox1* and *OsGA2ox6*. In contrast, the increase in *OsGA2ox3* transcripts observed in *m57–2* and decrease in *OsGA2ox3* transcripts observed in *m57–1* were not due to feedback mechanism (Fig. 4b). By scanning the promoter region of *OsGA2ox3*, we found that a *cis*-element was the same as the CArG-box in *D14* promoter region, bound by OsMADS57 protein (Guo et al. 2013a). Data from EMSA and dual-luciferase reporter assays in rice protoplasts proved that *OsGA2ox3* is a direct target of OsMADS57 (Fig. 5e). Deficiency of OsMADS57 protein in *m57–2* relieved the inhibition of OsMADS57 on *OsGA2ox3*, resulting in reduced bioactive GA levels, followed by the reduced plant height of *m57–2*. Surprisingly, *m57–1*, containing truncated OsMADS57 protein lacking an intact C-terminus (Guo et al. 2013a), also showed semi-dwarf phenotype (Fig. 1), which may result from the down-regulation of *OsGA20ox2* by other regulators because mutation in *OsGA20ox2* led to reduced GA levels and semi-dwarf phenotype (Spielmeyer et al. 2002; Sasaki et al. 2002). In addition, *m57–2* and *m57–1* had increased response to GA compared to wild type (Fig. 3c and d). It is intriguing that, as the suppressor of GA signaling pathway, the transcription of *SLR1* and *SLRL1* was promoted in two mutants (Fig. 4c). Although accumulation of SLR1 and SLRL1 limits GA responsiveness, post-transcriptional modification may also determine protein levels (Badodi et al. 2015). SLR1 and SLRL1 protein accumulation in vivo need to be further verified. These results prove that semi-dwarf phenotype in *osmads57* mutants result from GA deficiency but not malfunction in GA signaling.

### OsMADS57 directly represses *EUI1* transcription to regulate internode elongation

GA signaling and metabolism are involved in the regulation of internode elongation (Fleet and Sun 2005; Vriezen et al. 2003). GA levels are determined by GA3ox and GA20ox that are involved in GAs biosynthesis (Hedden and Thomas 2012), but also GA2ox that deactivates GAs (Thomas et al. 1999). *EUI1* is also involved in GA deactivation reaction by catalyzing 16α, 17-epoxidation reaction of GA_4_, GA_9_ and GA_12_ (Zhu et al. 2006; Ma et al. 2006). In *eui1* mutant, the uppermost internode is significantly elongated with enhanced panicle exsertion, and accumulates higher levels of GA than wild type. By contrast, the lines overexpressing *EUI1* exhibited significantly dwarf phenotype (Luo et al. 2006; Zhu et al. 2006). Data showed that both *m57–2* and *m57–1* had reduced elongation in the uppermost internode and defected in panicle exsertion compared to that of wild type (Fig. 2). In addition, the expression of *EUI1* was enhanced in *m57–2* but reduced in *m57–1* (Fig. 4b). MADS-box proteins function by regulating the expression of target genes via binding to CArG-box motifs (Gramzow and Theissen 2010; Tang and Perry 2003). Through browsing the promoter region of *EUI1*, a potential binding site of OsMADS57 was found. Analysis of LUC activity and EMSA confirmed the hypothesis that OsMADS57 directly regulated the expression of *EUI1*. In *m57–2*, the suppression of OsMADS57 on *EUI1* was abolished, which caused higher transcript levels of *EUI1* and thus reducing bioactive GA levels. Hence, internode elongation and panicle exsertion were hindered in *m57–2* plants. In addition, HOX12 can directly promote *EUI1* expression (Gao et al. 2016). Expectedly, the expression of *HOX12* was also greatly enhanced in *m57–2* plants. Therefore, panicle exsertion progress may not be regulated by OsMADS57 alone, but other protein factors synergisticly regulated this development process in combination with OsMADS57. However, *m57–1* plants also exhibited semi-dwarf phenotype with reduced uppermost internode elongation and defected panicle exsertion (Figs. 1 and 2). Previous research reported that overexpression of *CYP714B1* or *CYP714B2*, which are highly expressed in the uppermost internode of adult plants, causes semi-dwarf phenotype by reducing GA activity; but *cyp714b1cyp714b2* double mutant shows a longer uppermost internode, thus exposing longer internode from the flag leaf sheath (Magome et al. 2013). *EUI1* (*CYP714D1*), *CYP714B1* and *CYP714B2,* belonging to CYP714 subfamilies, likely co-modulate GA-mediated growth and development. We suppose that, as a compensation mechanism, inhibition of *EUI1* expression may cause the up-regulation of homologous genes, which led to shorter internode and panicle enclosure. In addition, the *m57–1* plants also showed increased tiller numbers (Fig. 1), which was consistent with previous study (Guo et al. 2013a). Generally, rice plants with more tillers tends to exhibit dwarf phenotype (Ishikawa et al. 2005; Qi et al. 2011; Dai et al. 2018). In rice, overexpressing genes encoding GA2-oxidase increases tiller numbers but inhibits stem elongation, which is coupled with GA deficiency (Lo et al. 2008). Accordingly, overexpression of *OsYABBY1* leads to reduced GA levels in rice plants, and overexpression lines exhibit semi-dwarf phenotype and more tillers (Dai et al. 2007). Also, *MOC1* overexpression lines display increased tiller numbers and reduced plant height (Li et al. 2003). Although there is a negative correlation between plant height and tiller numbers, the specific molecular mechanisms is not clear. It is worth noting that expansins regulate cell size/elongation via cell wall expansion (Magneschi et al. 2009; Vriezen et al. 2003). *OsEXPA4* overexpressors have enhanced stem elongation via affecting cell sizes (Choi et al. 2003). We speculate that reduced stem length in *m57–2* and *m57–1* plants is associated with down-regulated expression of expansin genes.

## Conclusions

**Fig. 7:**
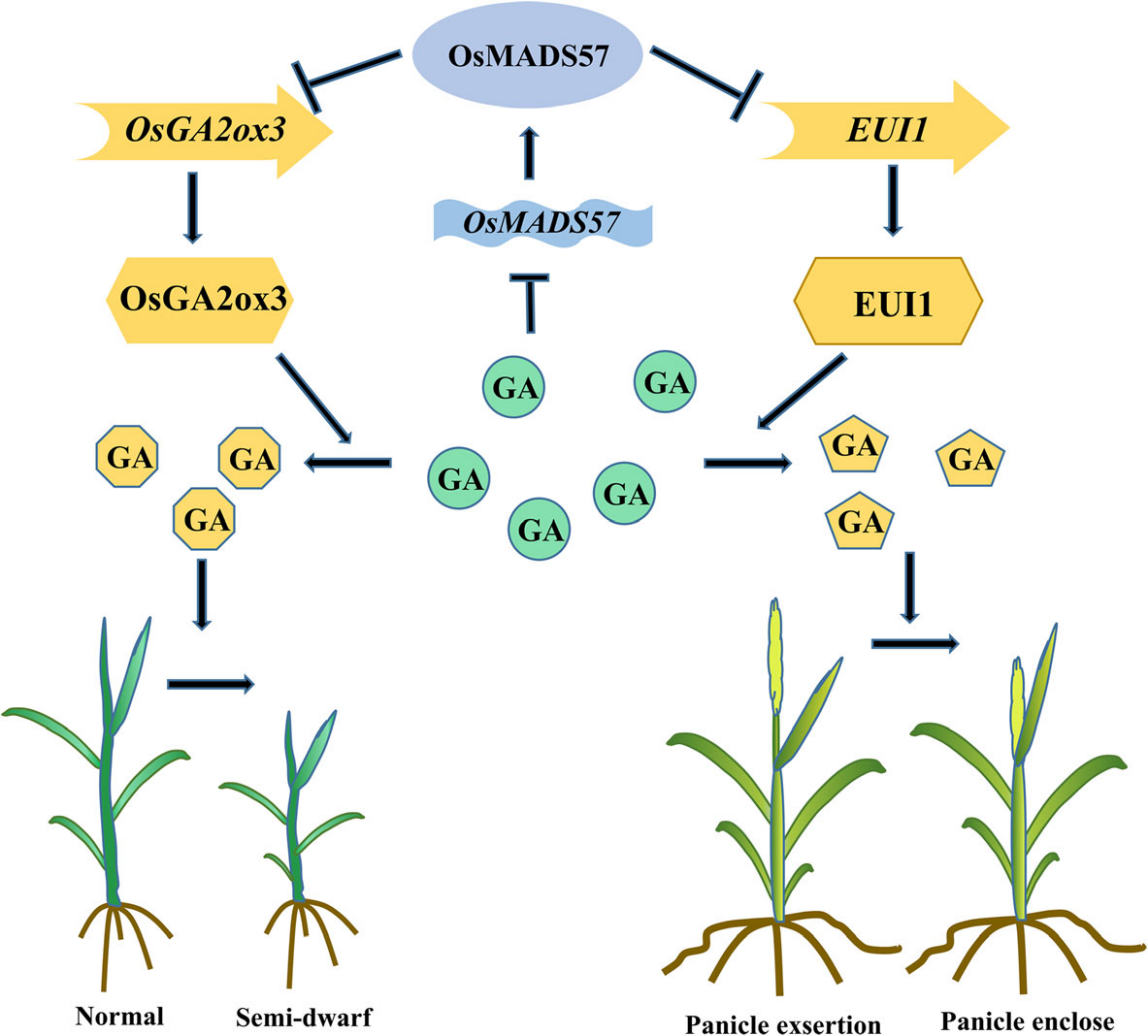
Model of OsMADS57 regulatory network through GA catabolic genes. The expression of *OsMADS57* is inhibited by GA_3_. And OsMADS57 suppresses the transcription of *OsGA2ox3* and *EUI1* that are involved in GA deactivation. Deficiency of OsMADS57 protein enhances the expression of *OsGA2ox3* and *EUI1*, which results in converting from bioactive GA to deactivated GA, thus reducing bioactive GA levels. Plants deficiency in bioactive GA shows panicle enclosure and semi-dwarf phenotype

In this work, OsMADS57 was shown to regulate internode elongation and plant height by directly repressing *OsGA2ox3* and *EUI1* expression. Deficiency of OsMADS57 protein promoted the expression of *OsGA2ox3* and *EUI1*, which enhanced the conversion of bioactive GA to deactivated GA, thus resulting in panicle enclosure and semi-dwarf phenotype (Fig. 7). The role of OsMADS57 in regulating plant growth provides an opportunity to improve grain yield by genetic manipulation to modulate stem elongation.

## Methods

### Plant materials and hormone treatment

The rice (*Oryza sativa*) mutants PFG_3A-15,619.R (*osmads57–2*) and PFG_3A-05432.L (*osmads57–1*) were obtained from RiceGE (the Rice Functional Genomics Express Database), in Pohang city, Korea. The mutants and their origin cultivar, *Oryza sativa ssp. japonica* cv Dongjin, were grown in a chamber with controlled temperature about 30 °C or in the fields during the suitable seasons. All the field management abides normal agricultural action. At maturity, the length of internode was recorded. For the phenotype analysis, the seeds of mutants and wild type were sterilized with 0.2% HgCl_2_ solution for 10 min, then washed five times with sterile distilled water and immersed in water for 2–3 d. These seeds were sown in half-strength Murashige and Skoog (1/2 MS) medium supplemented with 10 μМ GA_3_ or PAC at 30 °C. The shoot length of the seeding was measured. For *OsMADS57* expression analysis, 7-day-old wild-type seedlings were treated with 50 μМ GA_3_ or 10 μМ PAC, and harvested at different time points for Q-PCR analysis. For the seed germination assays, the sterilized seeds were placed on sterile filter paper moistened with double sterilized H_2_O and germinated in the dark. The germination rates were recorded each day.

### Total RNA extraction and quantitative PCR analysis

Total RNA was extracted from shoots of mutants and wild-type plants using TRIzol reagent (TaKaRa). RNA was used to synthesize cDNA using a PrimeScript™ RT reagent Kit from TaKaRa (code: RR047A). Q-PCR was performed on Bio-Rad CFX96 instrument with GoTaq qPCR Master Mix reagent (Promega) according to manufacturer’s instructions. The gene *Actin1* as rice housekeeping gene was used for an internal reference. Each analysis includes three biological repeats and three technical replicates. Primers used for Q-PCR are listed in Additional file 5: Table S1.

### Dual-luciferase reporter assays

For dual-luciferase reporter assay, the coding sequence (CDS) of *OsMADS57* was amplified by PCR and inserted into the pGreenII 62-SK vector for generating the effector. To generate reporter constructs, the promoter fragments of *EUI1* or *OsGA2ox3* were inserted into the pGreenII 0800 vector, respectively. The *Renilla luciferase* (*REN*) gene under the control of the *35S* promoter in the pGreenII 0800 vector was used as an internal control (Hellens et al. 2005). Empty pGreenII 0800 vector was used as the control. Protoplasts were prepared from rice shoots and transfected using a polyethylene glycolcalcium-mediated method followed by a 20-h incubation to allow transient expression (Zhang et al. 2011). Firefly LUC and REN activities were measured with a dual-luciferase reporter assay kit (Promega). The relative ratio of LUC to REN was calculated to represent the expression of reporter genes. Primers used for these constructs are listed in Additional file 5: Table S1.

### Electrophoretic mobility shift assays (EMSAs)

To test the binding activity of OsMADS57 protein, the full-length coding sequence of *OsMADS57* was cloned into pET32a (+) and transformed into BL21 (DE3). The OsMADS57 recombinant protein was purified using His60 Ni Superflow Resin. Oligonucleotide probes containing CArG motifs were synthesized and labeled with using a Biotin 3′End DNA Labeling Kit (Thermo Scientific). Unlabeled probe was used for competitive reactions. EMSA was performed using a LightShift® Chemiluminescent EMSA Kit (Thermo). Probe sequences are shown in Additional file 5: Table S1.

### Yeast two-hybrid

For yeast two-hybrid (Y2H) analysis, the CDS for *OsMADS57* and *SLR1* were cloned into the pGBKT7 and pGADT7 vectors, respectively. Detecting the interaction between OsMADS57 and SLR1 in yeast was performed. The yeast two-hybrid assay was conducted following the manufacturer’s instructions (Clontech). Primers used for these constructs are listed in Additional file 5: Table S1.

### Quantification of endogenous GA

For GA quantification, the shoots of two-week-old wild type and mutants plants were harvested and used for measurement of GA_3_ by a liquid chromatography-MS system (UPLC/Quattro Premier XE; Waters) according to the protocol (Kojima et al. 2009).

### Accession numbers

Sequence data from this article can be found in RICEGE or GenBank/EMBL databases under the following accession number: *OsMADS57* (Os02g0731200), *EUI1* (Os05g0482400), *OsGA2ox3* (Os01g0757200), *SLR1* (Os03g0707600), *SLRL1* (Os01g0646300), *HOX12* (Os03g0198600), *OsNAC2* (Os04g0460600), *OsYABBY4* (Os02g0643200), *OsPIL1* (Os03g0782500), *OsGA20ox1* (Os03g0856700), *OsGA20ox2* (Os01g0883800), *OsGA2ox6* (Os04g0522500), *CPS1* (Os02g0278700), *KO2* (Os06g0570100), *OsGA2ox1* (Os05g0158600), *OsGA3ox1* (Os05g0178100), *OsEXPA2* (Os01g0823100), *OsGA3ox2* (Os01g0177400), *OsEXPA1* (Os04g0228400), *OsEXPA3* (Os05g0276500), *OsEXPA4* (Os05g0477600), *OsEXPL1* (Os03g0132200) and *OsEXPB3* (Os10g0555900).

## Additional files

Additional file 1: **Figure S1.** *m57–2* and *m57–1* mutants exhibit delayed flowering phenotype. **a** Schematic diagram indicating the T-DNA insertion site in genomic region in *m57–2*. **b** PCR analyse the genotype of *m57–2* T_2_ seedlings. **c** Sequencing result for identifying of insertion site in the genomic region of *m57–2*. **d** Comparison of flowering between mutants and wild type. Arrows indicate flowering panicles. Bars = 10 cm. **e** Magnification of the boxed region in (**d**). Bars = 5 cm. **f** Quantitation of the seed germination rate of wild type and mutants lines. DJ, wild type; *m57–2*, *osmads57–2*; *m57–1*, *osmads57–1*. Three independent experiments were conducted with similar results. (TIF 6740 kb)

Additional file 2: **Figure S2.** *m57–2* and *m57–1* displayed reduced seed setting rate. **a** Flag leaf of wild type and mutants. Bars = 5 cm. **b** Flag leaf length of wild type and mutants at maturity. **c** Quantification of seed setting rate in wild type and mutants. **d** Comparison of grains between wild type and mutants, indicating blight grain rate increased in mutants. Bars = 2 cm. DJ, wild type; *m57–2*, *osmads57–2*; *m57–1*, *osmads57–1*. Three independent experiments were conducted with similar results. The data are means± SD (*n* = 10). Error bars indicate SD. The statistical significance of the measurements was determined by Student’s *t*-test. Asterisks indicate the significant difference between *osmads57* and wild type. (*t*-test, ^*^ *P* < 0.05, ^**^ *P* < 0.01 or ^***^ *P* < 0.001). (TIF 5617 kb)

Additional file 3: **Figure S3.** Shoot length of *m57–2*, *m57–1* and wild type under GA or PAC treatment at different time point. **a** Shoot length of wild type and mutants with GA treatment. **b** Shoot length of wild type and mutants with PAC treatment. DJ, wild type; *m57–2*, *osmads57–2*; *m57–1*, *osmads57–1*. Three independent experiments were conducted with similar results. The data are means± SD (*n* = 20). Error bars indicate SD. (TIF 938 kb)

Additional file 4: **Figure S4.** Physical interaction analysis between OsMADS57 and SLR1 in yeast. **a** Schematic representation of various combination between different constructs. **b** Yeast two-hybrid analysis the interaction of SLR1 and OsMADS57, and no interaction was observed between SLR1 and OsMADS57. Positive control, pGADT7-T (SV40 large T antigen)/pGBKT7–53 (murine p53). (TIF 5207 kb)

Additional file 5: **Table S1.** Primer and probe sequences used in this study. (XLSX 12 kb)

Additional file 6: **Data Set S1.** The up-regulated or down-regulated genes with│log2 ratio│≧1 in *osmads57–2* plants from the RNA-seq data. (XLSX 558 kb)

## Data Availability

The datasets used or analysed in this study are included in the article and its additional files.

## Abbreviations

CPS: *Ent*-copalyl diphosphate synthase
EMSAs: Electrophoretic mobility shift assays
*EUI1*: *ELONGATED UPPERMOST INTERNODE1*
GA: Gibberellin
GA20ox: GA 20-oxidase
GA2ox: GA 2-oxidase
GA_3_: Gibberellic acid
GA3ox: GA 3-oxidase
GARE: GA-responsive elements
*GID1*: *GIBBERELLIN INSENSITIVE DWARF1*
HD-ZIP: Homeodomain-leucine zipper
KAO: *Ent*-kaurenoic acid oxidase
KO: *Ent*-kaurene oxidase
KS: *Ent*-kaurene synthase
MS: Murashige and Skoog
PAC: Paclobutrazol
Q-PCR: Quantitative PCR
RNA-seq: RNA sequencing
SLR1: SLENDER1
WT: Wild type
Y2H: Yeast two-hybrid
