# Supplementary figures and images for: Rice transcription factor OsMADS57 regulates plant height by modulating gibberellin catabolism
Source: Rice (N Y). 2019 May 28;12:38. doi: 10.1186/s12284-019-0298-6 (PMC6538746; doi:10.1186/s12284-019-0298-6)

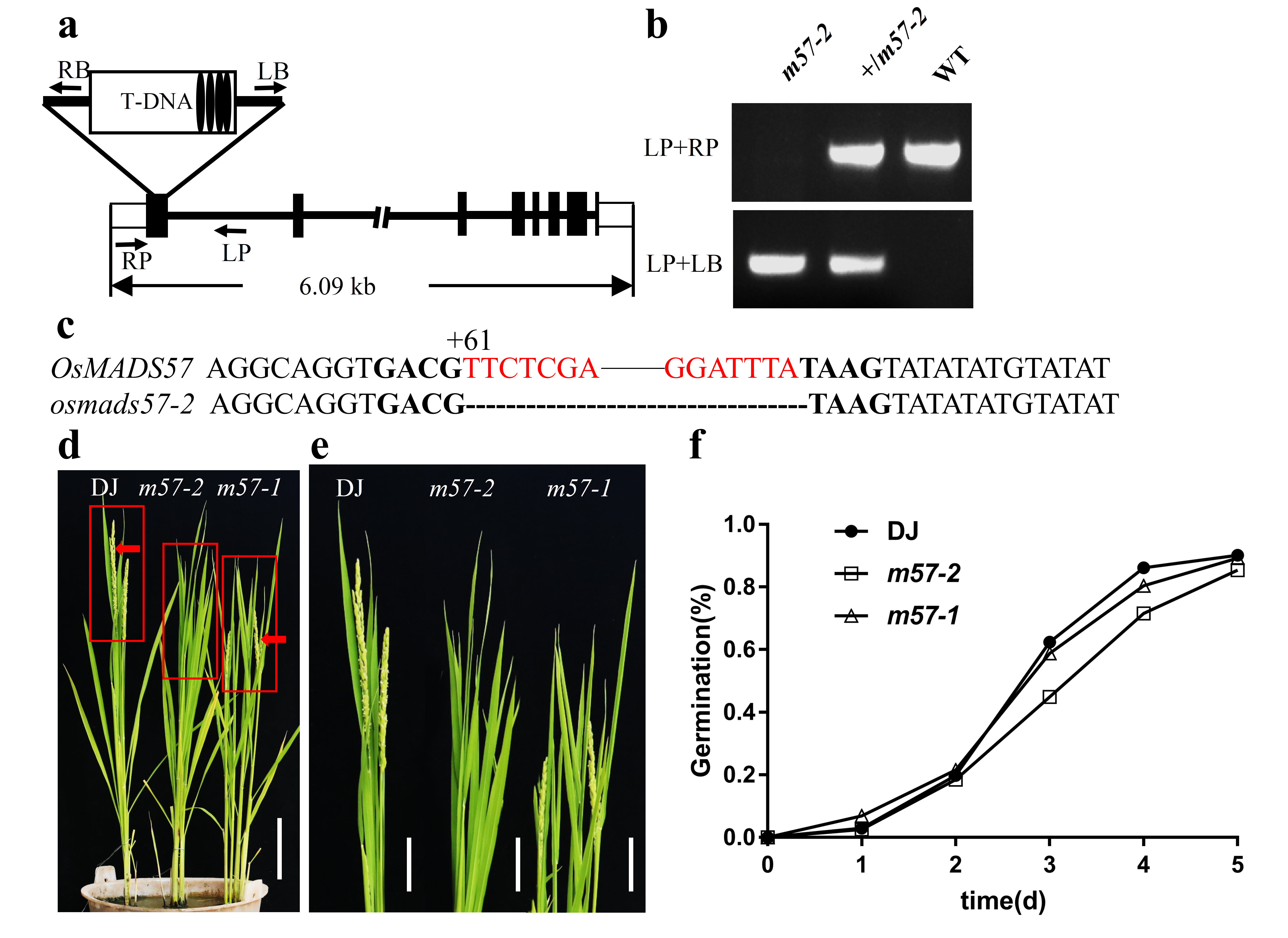

Supplement: Supplementary file 1 — Figure S1. m57–2 and m57–1 mutants exhibit delayed flowering phenotype. a Schematic diagram indicating the T-DNA insertion site in genomic region in m57–2. b PCR analyse the genotype of m57–2 T2 seedlings. c Sequencing result for identifying of insertion site in the genomic region of m57–2. d Comparison of flowering between mutants and wild type. Arrows indicate flowering panicles. Bars = 10 cm. e Magnification of the boxed region in (d). Bars = 5 cm. f Quantitation of the seed germination rate of wild type and mutants lines. DJ, wild type; m57–2, osmads57–2; m57–1, osmads57–1. Three independent experiments were conducted with similar results. (TIF 6740 kb) [file 12284_2019_298_MOESM1_ESM.tif]

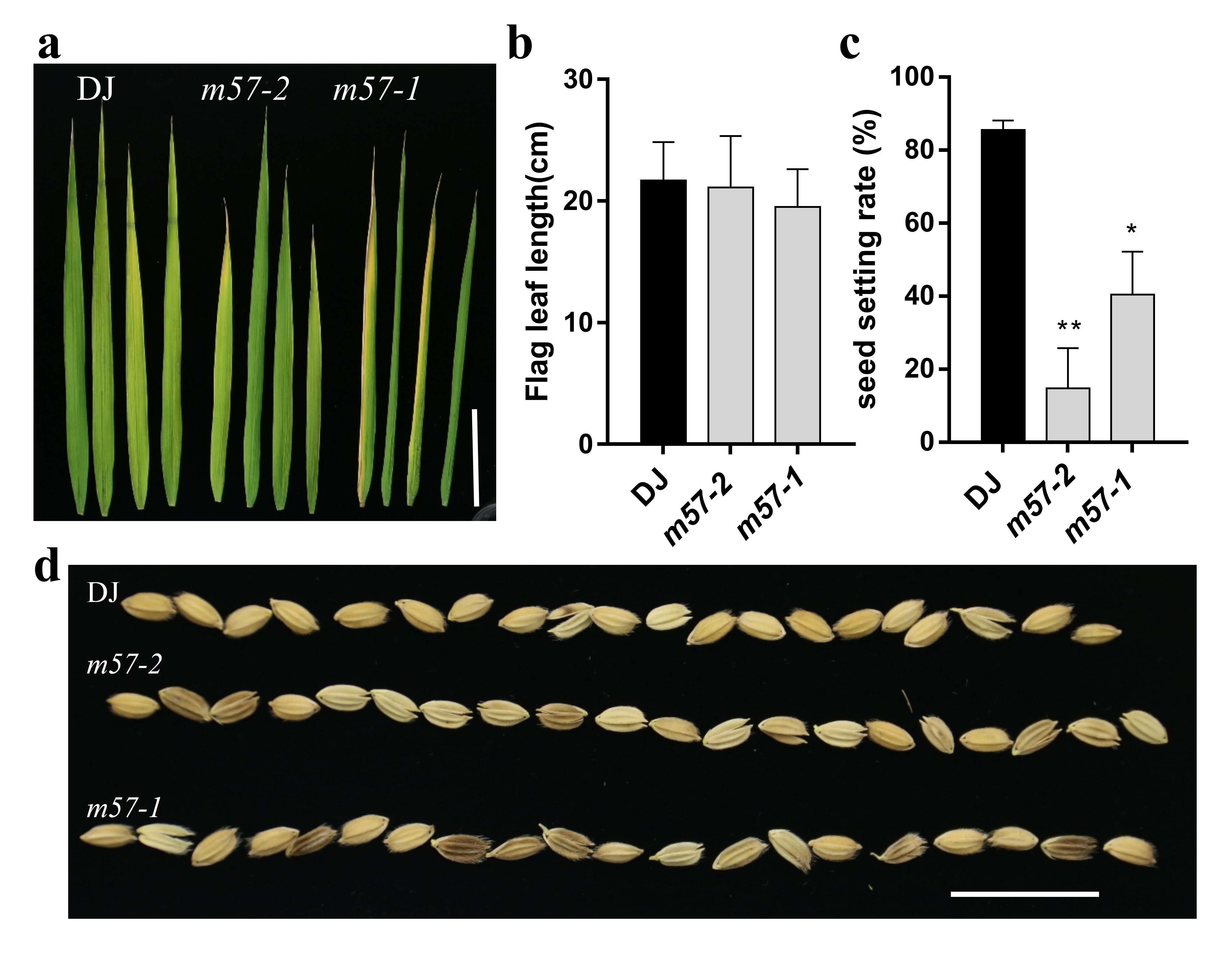

Supplement: Supplementary file 2 — Figure S2. m57–2 and m57–1 displayed reduced seed setting rate. a Flag leaf of wild type and mutants. Bars = 5 cm. b Flag leaf length of wild type and mutants at maturity. c Quantification of seed setting rate in wild type and mutants. d Comparison of grains between wild type and mutants, indicating blight grain rate increased in mutants. Bars = 2 cm. DJ, wild type; m57–2, osmads57–2; m57–1, osmads57–1. Three independent experiments were conducted with similar results. The data are means± SD (n = 10). Error bars indicate SD. The statistical significance of the measurements was determined by Student’s t-test. Asterisks indicate the significant difference between osmads57 and wild type. (t-test, * P < 0.05, ** P < 0.01 or *** P < 0.001). (TIF 5617 kb) [file 12284_2019_298_MOESM2_ESM.tif]

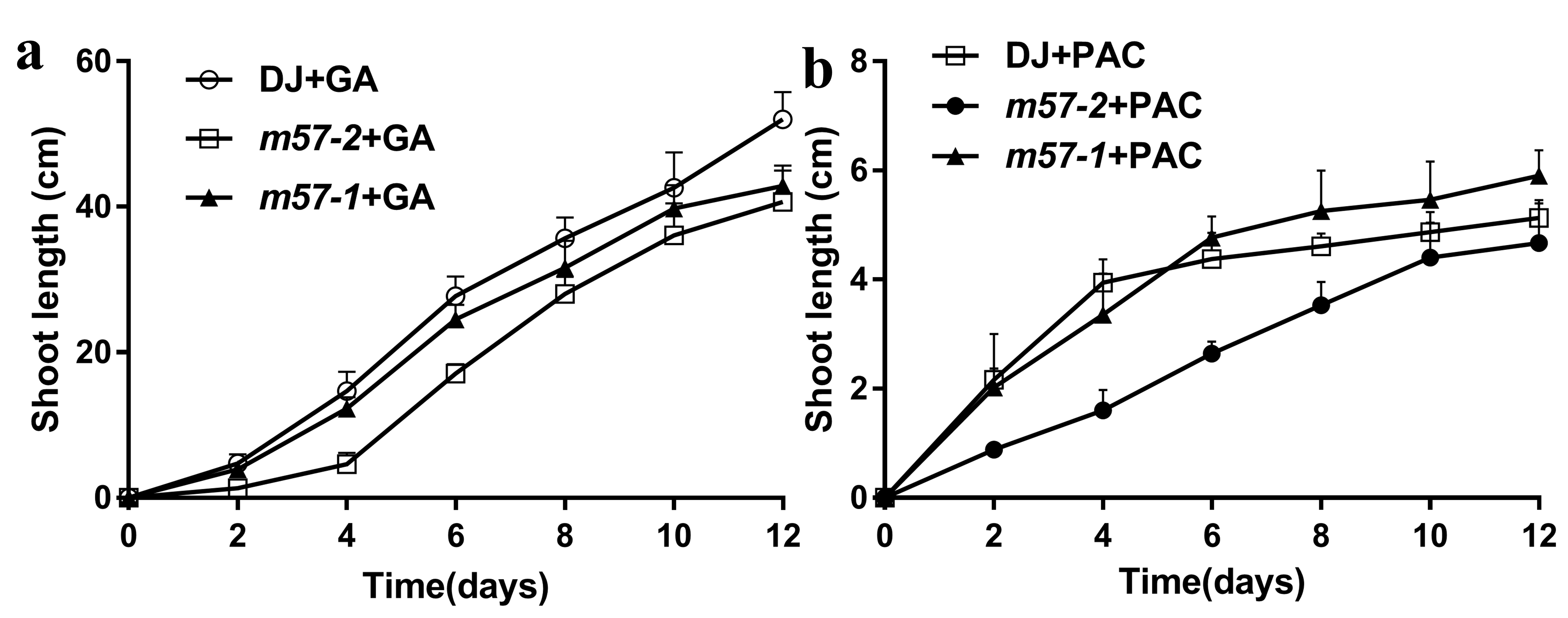

Supplement: Supplementary file 3 — Figure S3. Shoot length of m57–2, m57–1 and wild type under GA or PAC treatment at different time point. a Shoot length of wild type and mutants with GA treatment. b Shoot length of wild type and mutants with PAC treatment. DJ, wild type; m57–2, osmads57–2; m57–1, osmads57–1. Three independent experiments were conducted with similar results. The data are means± SD (n = 20). Error bars indicate SD. (TIF 938 kb) [file 12284_2019_298_MOESM3_ESM.tif]

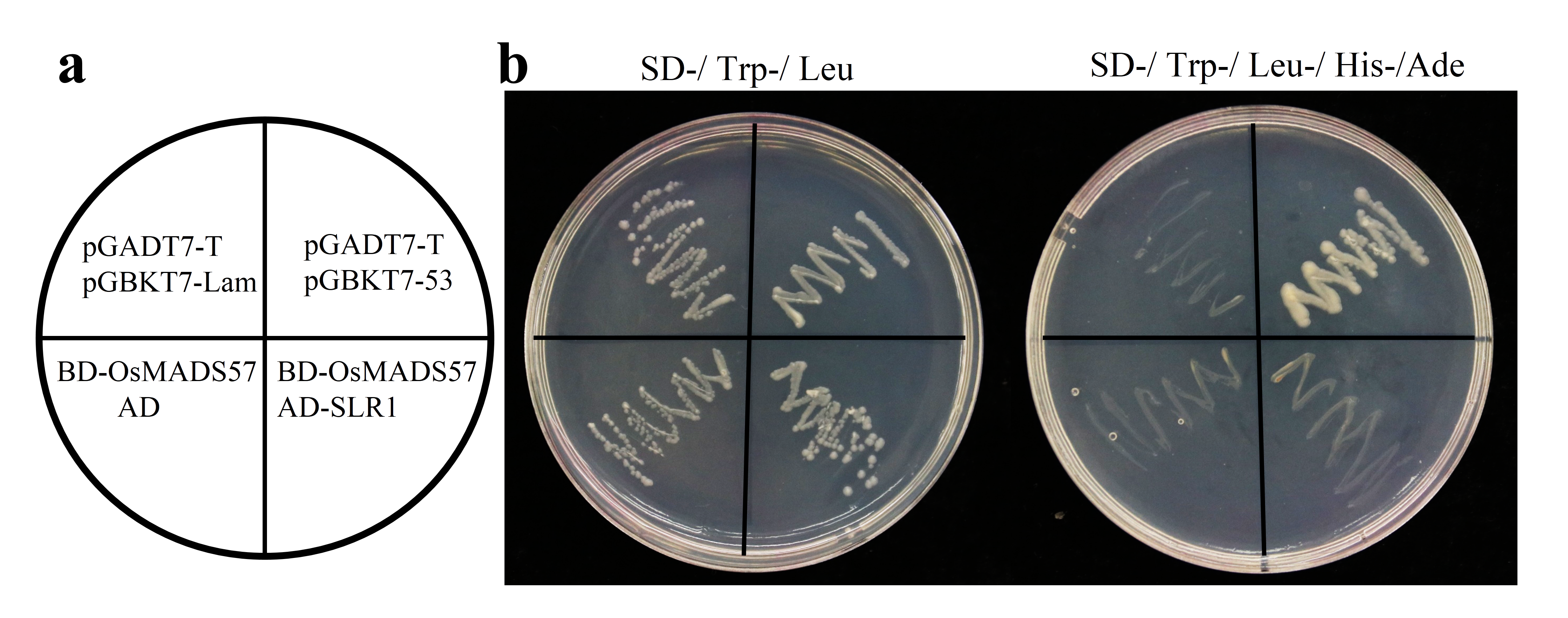

Supplement: Supplementary file 4 — Figure S4. Physical interaction analysis between OsMADS57 and SLR1 in yeast. a Schematic representation of various combination between different constructs. b Yeast two-hybrid analysis the interaction of SLR1 and OsMADS57, and no interaction was observed between SLR1 and OsMADS57. Positive control, pGADT7-T (SV40 large T antigen)/pGBKT7–53 (murine p53). (TIF 5207 kb) [file 12284_2019_298_MOESM4_ESM.tif]
